# Surface Strain Evolution and Cracking Behavior of Concrete Under Non-Uniform Corrosion-Induced Expansion Monitored by Distributed Fiber Optics

**DOI:** 10.3390/s26154889

**Published:** 2026-08-03

**Authors:** Qiangqiang Ma, Liang Fan, Yongjun Zhang, Baorong Hou

**Affiliations:** 1 School of Civil Engineering, Qingdao University of Technology, Qingdao 266520, China; 2 State Key Laboratory of Advanced Marine Materials, Shandong Key Laboratory of Marine Environmental Corrosion and Bio-Fouling, Institute of Oceanology, Chinese Academy of Sciences, Qingdao 266071, China

**Keywords:** distributed optical fiber sensing, non-uniform corrosion-induced cracking, biochar, cover thickness, two-stage strain evolution

## Abstract

Cover cracking induced by steel corrosion is a critical issue governing the durability degradation of reinforced concrete structures in marine environments. The crack initiation and propagation processes dominated by non-uniform rust expansion stress fields urgently require high-resolution continuous monitoring techniques. In this study, based on the principle of Rayleigh backscattering, distributed optical fibers were embedded along the upper surface of specimens to conduct in situ monitoring of surface strain in concrete. The effects of specimen length, biochar content, cover thickness, and rebar diameter were systematically investigated. The results indicate that the surface strain evolution follows a two-stage pattern—a slow growth stage followed by a rapid rise stage—corresponding respectively to the early-stage filling of interfacial pores and stress accumulation, and the later-stage propagation of macroscopic cracks. Increasing specimen length significantly amplifies the spatiotemporal non-uniformity of strain, characterized by “locally high peak strains but low overall mean values,” with the onset time of strain surges differing by more than 50 h across different cross-sections. The incorporation of 0.5% biochar reduces the average strain by approximately 17% and delays crack initiation to 260 h. Increasing cover thickness from 25 mm to 40 mm exhibits the most pronounced inhibitory effect, achieving a 39% reduction in strain and delaying crack initiation to 320 h, primarily attributed to the extended chloride transport path and enhanced hoop confinement stiffness. Reducing rebar diameter from 20 mm to 12 mm decreases the peak strain to 79% of that of the standard specimen, owing to reduced rust product volume and increased relative cover thickness. The macro-cell effect driven by chloride concentration gradient transition zones is identified as a key factor governing crack initiation locations. Theoretical crack widths derived from strain integration of optical fiber data are slightly lower than measured values, yet the overall trends remain consistent. This study provides a quantitative basis for continuous monitoring and durability assessment of corrosion-induced cracking in marine environments.

## 1. Introduction

Corrosion of reinforcing steel is one of the primary causes of cracking in reinforced concrete (RC) structures. Once corrosion-induced cracks initiate and propagate through the concrete cover to the exposed steel rebar surface, they provide preferential pathways for the ingress of aggressive agents, including chloride ions, oxygen, and moisture. This process further accelerates reinforcement corrosion [1] and forms a self-sustaining deterioration cycle, ultimately leading to a significant reduction in structural durability. Consequently, the initiation and spatial distribution of corrosion-induced cracks determine the cracking pattern of the concrete cover, whereas the subsequent evolution of crack width directly governs the long-term durability and service life of reinforced concrete structures.

However, corrosion-induced cracking exhibits pronounced spatiotemporal heterogeneity. Owing to the combined effects of concrete porosity gradients, localized chloride accumulation, and macro-cell corrosion along the reinforcing steel, cracks rarely develop uniformly along the longitudinal direction of the reinforcement. Instead, cracking preferentially initiates at mechanically vulnerable locations and subsequently propagates asynchronously at different positions along the reinforcement [2]. Such intrinsic spatial discreteness and temporal asynchrony considerably complicate the monitoring and quantitative characterization of crack evolution, particularly under long-term marine exposure conditions.

To investigate the evolution of corrosion-induced strain and cracking, a variety of monitoring techniques have been developed [3,4]. Conventional point-based sensing techniques, including electrical resistance strain gauges, vibrating-wire strain gauges, and laser displacement sensors, have been widely employed for localized measurements of concrete surface strain and crack width because of their high measurement accuracy [5,6]. Nevertheless, the limited number of sensing locations prevents these techniques from providing continuous spatial information over the entire structural surface. Since the initiation of corrosion-induced cracks is inherently random and spatially non-uniform, point sensors are likely to miss the actual crack initiation locations and therefore fail to capture the complete crack propagation process.

Electrochemical methods, such as linear polarization resistance (LPR), electrochemical noise (EN), and electrochemical impedance spectroscopy (EIS), estimate the corrosion state of reinforcing steel by measuring electrochemical parameters, including corrosion current density [7,8]. Although these techniques are effective for evaluating corrosion activity, the obtained information is indirect and cannot directly characterize the evolution of concrete surface strain or crack propagation. In the field of nondestructive evaluation, ultrasonic testing, ground-penetrating radar (GPR), and acoustic emission (AE) techniques have also been widely applied to detect internal defects and crack signals within concrete. However, AE is incapable of quantitatively determining crack width, while the interpretation of GPR data is often computationally demanding and time-consuming. Optical full-field measurement techniques, particularly digital image correlation (DIC), enable high-resolution measurements of surface deformation and crack distribution by tracking surface displacement fields [9]. Nevertheless, under long-term marine environments characterized by high humidity and salt spray, deterioration of the speckle pattern substantially reduces measurement stability, thereby limiting the applicability of DIC for continuous long-term monitoring. X-ray computed tomography (X-ray CT) can reconstruct the three-dimensional morphology of corrosion products and internal cracks with excellent spatial resolution. However, the high equipment cost, limited specimen size, and complex testing procedures restrict its application to intermittent inspections rather than continuous monitoring throughout the entire corrosion process.

Recent advances in remote inspection and automated crack detection techniques have further expanded the capability of structural health monitoring. Image-based approaches, including digital image correlation, unmanned aerial vehicle (UAV)-based inspection, and deep learning-assisted crack recognition [10], have demonstrated promising performance in automated crack localization and quantitative assessment of concrete structures [11,12]. For example, automated crack detection methods based on convolutional neural networks and image processing techniques have achieved efficient identification of surface cracks, while UAV-based inspection provides a flexible solution for large-scale infrastructure assessment [13,14]. However, these approaches mainly rely on surface visual information and are generally more suitable for periodic inspection after damage occurrence rather than continuous monitoring of the internal damage evolution process. Therefore, sensing technologies capable of providing continuous strain measurements with high spatial resolution, such as distributed optical fiber sensing, remain essential for capturing the initiation and propagation mechanisms of corrosion-induced cracking.

Furthermore, existing monitoring techniques suffer from inherent limitations in spatial continuity, temporal continuity, or quantitative accuracy, making it difficult to continuously monitor the entire process of corrosion-induced crack initiation and propagation under non-uniform corrosion conditions with high spatial resolution. More importantly, the initiation time, propagation rate, and crack-width evolution at different locations have not yet been systematically quantified. Consequently, the mechanisms governing the heterogeneous spatial distribution of corrosion-induced cracking remain insufficiently understood.

In this study, a distributed optical fiber sensing (DOFS) system based on Rayleigh backscattering was employed to continuously monitor the surface strain evolution of reinforced concrete specimens subjected to electrically accelerated corrosion over a monitoring period of 350 h. Distributed optical fibers were embedded along the upper surface of the specimens to systematically investigate the influences of specimen length, biochar incorporation, concrete cover thickness, and reinforcing bar diameter on the initiation and propagation of corrosion-induced cracking. The temporal evolution of surface strain was analyzed to identify the characteristic stages of corrosion-induced deformation and to quantify the effects of different design parameters on crack-width development. Furthermore, by combining the measured strain field with chloride concentration distributions and crack-width measurements, the governing factors responsible for the heterogeneous spatial distribution of corrosion-induced cracks under non-uniform corrosion conditions were elucidated. The findings provide valuable experimental evidence for the continuous monitoring and durability assessment of corrosion-induced deterioration in reinforced concrete structures exposed to marine environments.

## 2. Materials and Methods

### 2.1. Raw Materials and Concrete Mix Proportions

The optical fiber used in this study was a GJHy-1B6b-0.9-G Haicui tight-buffered optical fiber manufactured by Beijing Xizhuo Information Technology Co., Ltd. (Beijing, China), with an outer diameter of 0.88 mm. HRB400 ribbed reinforcing steel bars with a nominal diameter of 20 mm were used as the reinforcement. Ordinary Portland cement (P.O 42.5) supplied by China United Cement Corporation (Weifang, China) was used as the cementitious material. Its chemical composition is listed in Table 1, and its properties comply with the requirements of GB 175–2007 Standard for Common Portland Cement [15]. ISO standard sand provided by Xiamen ISO Standard Sand Co., Ltd. (Xiamen, China). was used as the fine aggregate. The particle size ranged from 0.08 to 2.0 mm, satisfying the requirements of GB/T 17671 [16]. Artificial seawater was prepared in accordance with IS 8770–1978 [17]. Deionized water was mixed with inorganic salts, including sodium chloride (NaCl), magnesium chloride (MgCl_2_), sodium sulfate (Na_2_SO_4_), calcium chloride (CaCl_2_), potassium chloride (KCl), sodium bicarbonate (NaHCO_3_), and potassium bromide (KBr), to simulate the ionic composition of natural seawater. The chemical composition of the artificial seawater is summarized in Table 2. A polycarboxylate-based high-range water-reducing admixture with a water reduction ratio of 30% and a solid content of 39.2% was employed. The biochar used in this study was SC-102 sawdust biochar, supplied by Pingdingshan Tannuo Environmental Protection Materials Co., Ltd. (Pingdingshan, China). Its main physical properties are presented in Table 3.

### 2.2. Specimen Preparation

A total of four experimental groups were designed in this study. The specimen-length group consisted of specimens C1, C2, and C3; the biochar group consisted of C2 and C4; the concrete cover thickness group consisted of C2 and C5; and the reinforcing bar diameter group consisted of C2, C6, and C7. The dimensions, constituent materials, reinforcement diameters, concrete cover thicknesses, and mix proportions of all specimens are summarized in Table 4. All mixtures were prepared with a constant water-to-cement ratio of 0.41. All specimens had rectangular cross-sections. Specimens C2 and C4 measured 70 mm × 70 mm × 300 mm, specimen C1 measured 70 mm × 70 mm × 100 mm, specimen C3 measured 70 mm × 70 mm × 500 mm, specimen C5 measured 100 mm × 100 mm × 300 mm, specimen C6 measured 62 mm × 62 mm × 300 mm, and specimen C7 measured 75 mm × 75 mm × 300 mm. Pure artificial seawater was used as the mixing water for all specimens. For specimen C4, 0.5 wt.% of the cement was replaced with sawdust biochar to prepare the biochar-modified concrete.

Reinforcing bars were cut to the required lengths and mechanically polished to remove the original surface rust before concrete casting. During specimen preparation, the distributed optical fiber was embedded longitudinally within the concrete cover at a depth of 5 mm below the upper surface of the specimen. The optical fiber was arranged perpendicular to the reinforcing bar at multiple locations to continuously monitor the surface strain evolution of the concrete cover induced by corrosion product expansion. After casting, the specimens were demolded and cured under standard conditions. Subsequently, an acrylic ring was bonded to the upper surface of each specimen using epoxy resin to form a reservoir for the electrolyte solution during the accelerated chloride migration test.

### 2.3. Determination of Free Chloride Concentration on the Reinforcing Steel Surface

To quantitatively characterize chloride accumulation on the reinforcing steel surface, the free chloride concentration was determined after 350 h of electrically accelerated corrosion. Following the procedure adopted in previous studies, concrete powder samples were collected by drilling vertically from the exposed upper surface of the specimen toward the embedded reinforcement. The drilling depth was controlled to approximately 25 mm, corresponding to the depth of the reinforcing steel surface (i.e., the concrete cover thickness), and the collected powder represented the chloride environment immediately adjacent to the reinforcement. Eleven sampling locations were uniformly distributed along the length of each specimen to ensure that the measured chloride concentration accurately represented the corrosion environment surrounding the reinforcing steel.

The free chloride concentration was determined using the water-soluble extraction method. Specifically, 4 g of mortar powder was mixed with 20 mL of deionized water and thoroughly stirred before being sealed and stored for 24 h. The suspension was subsequently filtered through filter paper, and 10 mL of the supernatant was collected and acidified with nitric acid at a volume ratio of 1:1. Potentiometric titration was then performed using a 0.05 mol/L AgNO_3_ solution. The volume of silver nitrate consumed at the equivalence point was recorded to calculate the free chloride concentration. All titration procedures were conducted in accordance with ASTM C1218/C1218M-15 [18].

### 2.4. Distributed Optical Fiber Sensing System

A distributed optical fiber sensing (DOFS) system based on optical frequency domain reflectometry (OFDR) was employed to measure the strain distribution along the optical fiber by tracking the spectral shift in Rayleigh backscattering [19]. The distributed optical fiber sensing system employed in this study is manufactured by Wuhan Haoheng Technology Co., Ltd. (Wuhan, China). When the optical fiber was subjected to external strain or temperature variation, changes in its internal microstructure caused a shift in the Rayleigh backscattering spectrum. The wavelength shift, Δλ, is related to the strain variation (Δε) and temperature variation (ΔT), as expressed in Equation (1) [4]

(1) Δλ=α⋅Δε+β⋅ΔT

where α is the strain sensitivity coefficient (approximately 1.2 pm/με at a wavelength of 1550 nm), and β is the temperature sensitivity coefficient (approximately 13.2 pm/°C). During the experiment, a temperature-compensation fiber was employed to eliminate the influence of temperature fluctuations, thereby enabling the extraction of pure strain information. The sensing system provided a spatial resolution of 0.64 mm, allowing localized strain concentrations induced by corrosion expansion on the concrete surface to be accurately identified.

The optical fiber was embedded longitudinally along the upper surface of the concrete specimen and deformed synchronously within the surrounding concrete. As corrosion products accumulated on the reinforcing steel, corrosion-induced expansion generated expansive stresses at the steel–concrete interface, which were subsequently transferred to the concrete cover. The resulting tensile strain on the concrete surface was transmitted to the embedded optical fiber, producing a measurable Rayleigh spectral shift and enabling continuous strain monitoring [19,20]. Strain data were acquired at 2 h intervals over a monitoring period of 350 h, providing continuous strain histories at all sensing locations along the fiber.

Compared with conventional point-based sensors, distributed optical fiber sensing enables continuous full-length strain measurements. When localized cracking occurred owing to concentrated corrosion-induced expansion, a sudden increase in fiber strain was observed at the corresponding location. By tracking the evolution and migration of strain peaks along the optical fiber, both the crack initiation location and subsequent propagation path could be identified. This technique therefore provides high-resolution experimental data for investigating the spatial heterogeneity and asynchronous evolution of corrosion-induced cracking in reinforced concrete subjected to non-uniform corrosion.

### 2.5. Accelerated Corrosion Test

To accelerate the electrochemical corrosion process of reinforcing steel under chloride exposure, an electrically accelerated corrosion method with a constant current was adopted. By applying a constant current density, chloride ions were driven towards the reinforcing steel under an electric field, thereby accelerating the breakdown of the passive film and the formation of corrosion products. To ensure a comparable current input per unit surface area of reinforcing steel, different current levels were applied according to specimen length. Based on previously reported current-density selection criteria for accelerated corrosion tests [21], constant currents of 9 mA, 27 mA, and 45 mA were applied to specimens with lengths of 100 mm, 300 mm, and 500 mm, respectively, thereby maintaining a consistent electrical input per unit surface area of reinforcement. The accelerated corrosion test serves as an effective experimental approach for investigating the evolution mechanism of corrosion-induced cracking within a reasonable experimental duration.

**Figure 1:**
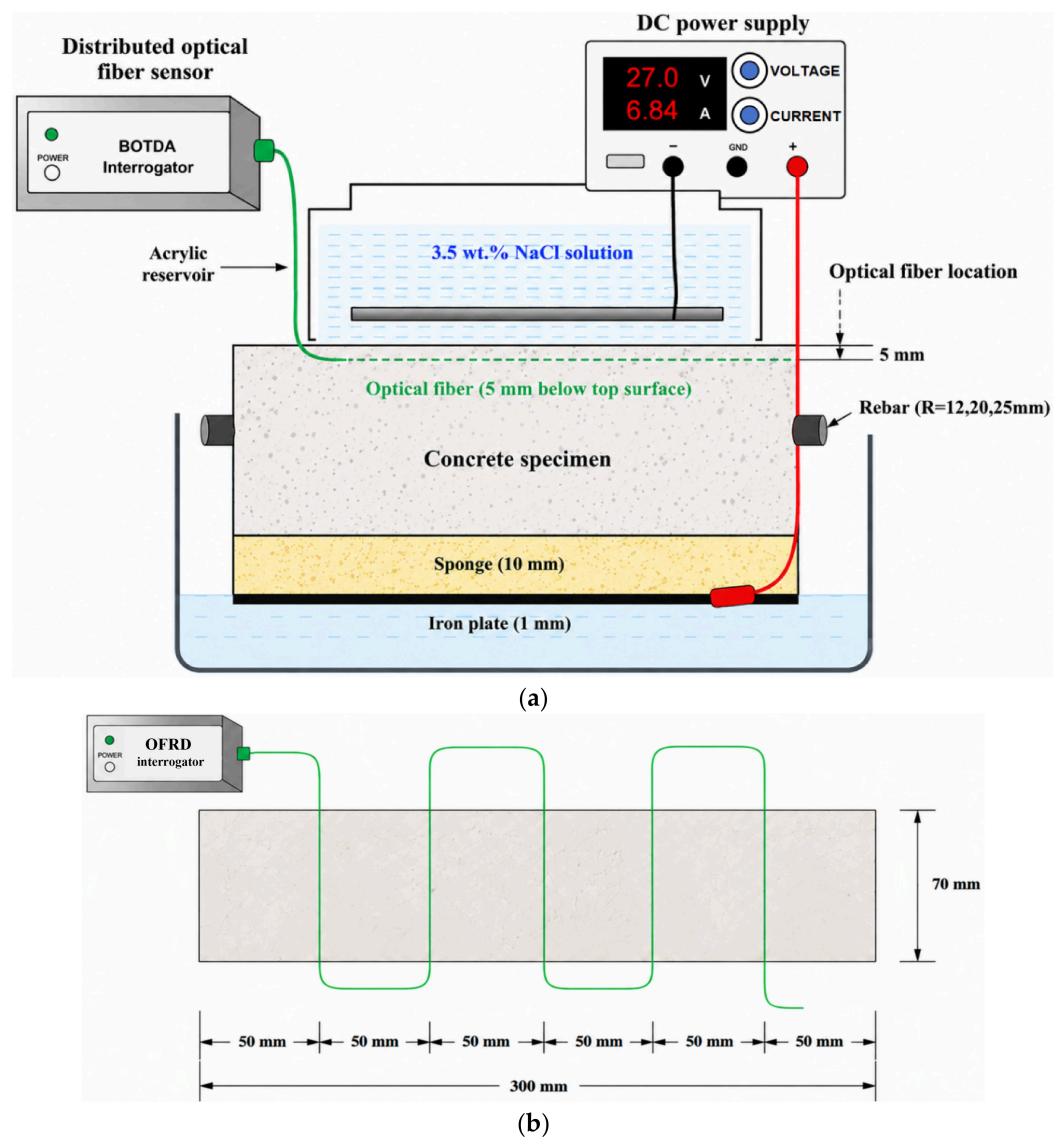
(**a**) Accelerated corrosion test setup, (**b**) fiber arrangement along the specimen.

During the corrosion test, a stainless-steel plate was placed in the water reservoir on the upper surface of the specimen and immersed in a 3.5 wt.% NaCl solution. The stainless-steel plate was connected to the positive terminal of the DC power supply. The bottom of the specimen was supported by a 10 mm thick sponge saturated with 3.5 wt.% NaCl solution. A 1 mm thick iron plate with the same dimensions as the specimen base was positioned beneath the sponge and connected to the negative terminal of the power supply. The corrosion-induced concrete surface strain was continuously monitored using the distributed optical fiber sensing system, as illustrated in Figure 1.

Distributed optical fibers were embedded perpendicular to the reinforcing steel at a depth of 5 mm below the upper concrete surface. The number and spacing of the embedded fibers were adjusted according to specimen length. Four fibers were embedded in the 100 mm specimens with a spacing of 20 mm, whereas five and six fibers were embedded in the 300 mm and 500 mm specimens with spacings of 50 mm and 71.4 mm, respectively (Figure 1). Each optical fiber provided continuous strain measurements at 108 sensing points. To characterize the strain evolution in the most severely cracked region, the ten sensing points exhibiting the highest strain values on each fiber were selected, and their arithmetic mean was calculated to represent the average surface strain at the crack location at each measurement interval. Monitoring began immediately after the current was applied and continued for 350 h, with a sampling interval of 2 h.

In this study, an independent distributed optical fiber was used exclusively for temperature compensation. The temperature compensation fiber was arranged along the side surface of the concrete specimen and maintained in close contact with the concrete surface. This arrangement ensured that the compensation fiber experienced essentially the same thermal environment and temperature variation as the optical fibers embedded inside the concrete. Unlike the embedded sensing fibers, the compensation fiber was intentionally located outside the corrosion-affected region and was therefore not subjected to corrosion-induced deformation or crack development.

### 2.6. Measurement of Concrete Crack Width

Concrete crack widths were determined using a combination of microscopic observation and quantitative measurement. A TESI handheld crack width gauge (Beijing Taixin Instrument Co., Ltd., Beijing, China) was employed to measure the crack width on the concrete surface. The instrument determines crack width through optical magnification combined with an internally calibrated measurement scale. During each measurement, the optical probe was positioned perpendicular to the crack surface, and multiple representative locations were selected at equal intervals along the crack length. The crack width at each location was recorded individually, and the arithmetic mean of all measurements was taken as the final crack width of the crack. The measurement system provided a spatial resolution of 0.01 mm, a typical measurement accuracy of ±0.02 mm, and a minimum detectable crack width of approximately 0.02 mm, which satisfied the requirements for microcrack measurements. The primary sources of measurement uncertainty included subjective identification of crack boundaries, surface roughness of the concrete, and non-uniform crack opening along the crack length. To minimize measurement errors, each crack was measured at least three times, and the average value was adopted. The repeatability error was generally maintained within 5%, demonstrating the good stability and reliability of the measurement method.

## 3. Results and Discussion

### 3.1. Effects of Different Parameters on Corrosion-Induced Surface Strain of Concrete

#### 3.1.1. Time-History Characteristics of Strain at the Optical Fiber with the Maximum Strain Response

**Figure 2:**
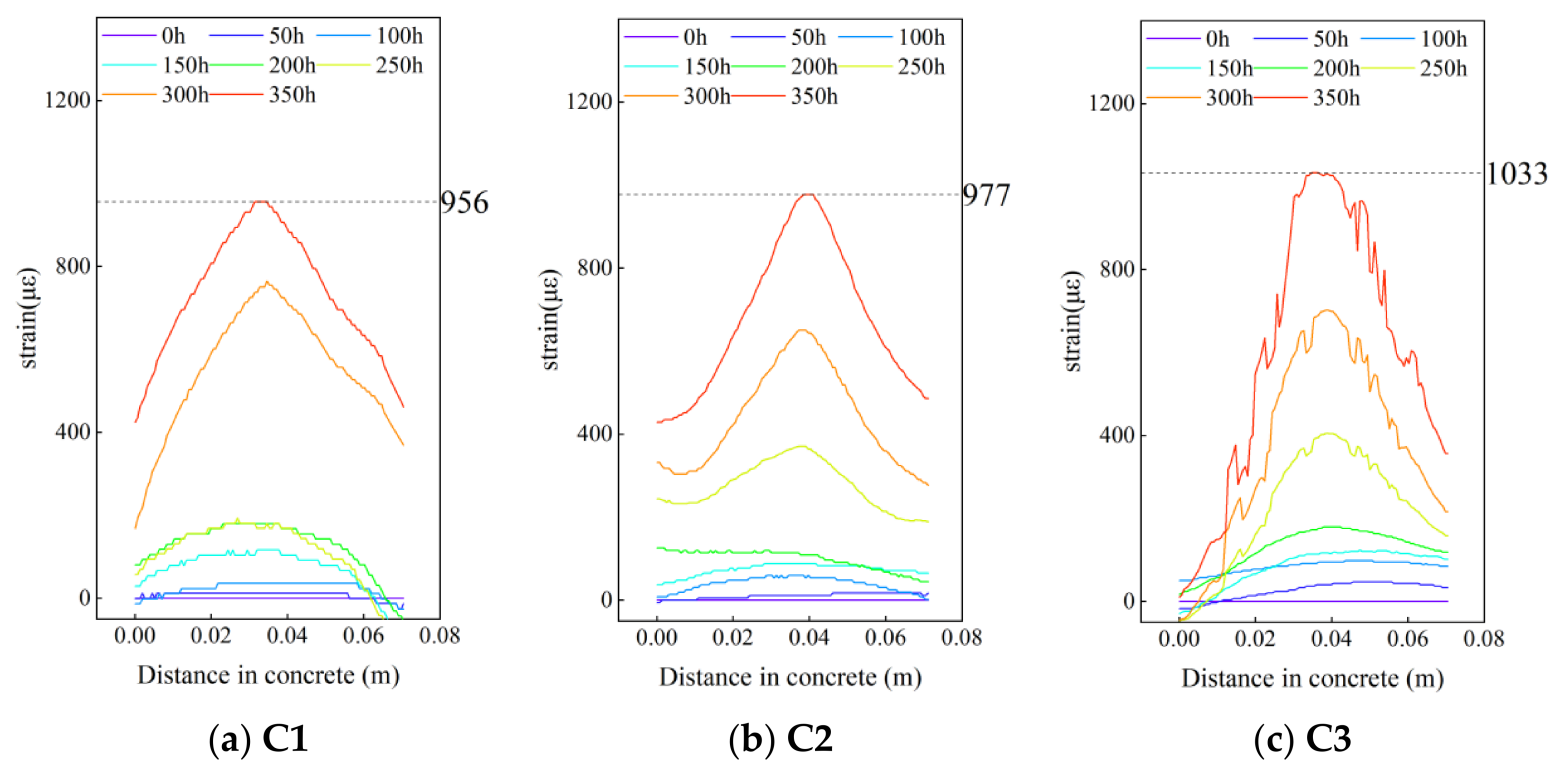

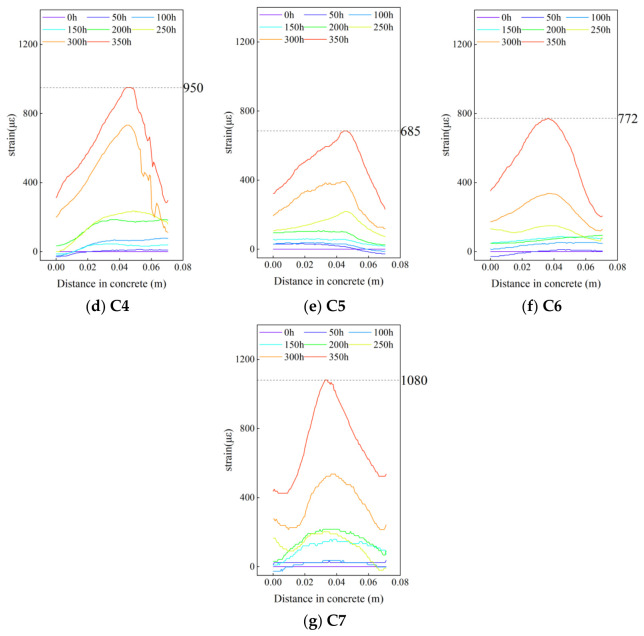
Strain-time curves of the maximum strain optical fiber along its length direction in each group of specimens C1 to C7.

To investigate the influence of different experimental variables on the evolution of corrosion-induced surface strain and crack development in concrete, the optical fiber exhibiting the maximum strain response in each specimen, which was embedded perpendicular to the reinforcing steel, was selected for analysis. The strain–time histories of all sensing points along the selected optical fiber were extracted. Figure 2a–g present the strain evolution at each sensing point of the optical fiber with the maximum strain response for specimens C1–C7, respectively. The strain distributions are plotted at 50 h intervals to clearly illustrate the evolution of strain concentration and its spatial distribution during the accelerated corrosion process. By comparing the overall evolution patterns of the seven groups, the effects of specimen length, biochar incorporation, concrete cover thickness, and reinforcing bar diameter on the development of localized strain can be preliminarily evaluated.

After 350 h of accelerated corrosion, the maximum strain measured by the optical fiber in the reference specimen (C2) reached 977 με. Increasing the specimen length to 500 mm (C3) intensified corrosion localization, resulting in a further increase in the peak strain to 1033 με, which was 1.06 times that of specimen C2. The incorporation of 0.5 wt.% biochar reduced the peak strain to 950 με, corresponding to approximately 97% of the value measured for C2. Increasing the concrete cover thickness to 40 mm (C5) significantly reduced the peak strain to 685 με, representing only 70% of that of C2. Similarly, reducing the reinforcing bar diameter to 12 mm (C6) decreased the peak strain to 772 με, corresponding to approximately 79% of that of C2. Among all investigated parameters, increasing the concrete cover thickness and reducing the reinforcing bar diameter exhibited the most pronounced suppression of surface strain development. Although these two parameters differ in terms of structural design, they share a common mechanical mechanism by substantially increasing the relative concrete cover thickness (c/d), where c and d denote the concrete cover thickness and reinforcing bar diameter, respectively. Increasing the c/d ratio from 1.25 to 2.00 by increasing the cover thickness, and to 2.08 by reducing the reinforcing bar diameter, effectively enhanced the circumferential confinement stiffness of the concrete cover against corrosion-induced expansion. Consequently, the peak circumferential tensile stress within the concrete cover decreased under the same corrosion-induced expansive force. Moreover, increasing the concrete cover thickness extended the stress transfer path from the steel–concrete interface to the concrete surface, thereby promoting stress redistribution and energy dissipation and effectively suppressing the rapid increase in surface strain [22,23].

A further comparison of the strain–time histories shown in Figure 2 reveals that specimens C4 (biochar), C5 (40 mm cover thickness), and C6 (12 mm reinforcing bar diameter) exhibited no obvious strain surge during the first 250 h of accelerated corrosion, whereas the reference specimen C2 had already entered the rapid strain-growth stage at approximately 250 h. These observations indicate that biochar incorporation, increased concrete cover thickness, and reduced reinforcing bar diameter not only effectively decreased the final strain magnitude but also significantly delayed crack initiation. It should be noted that the evolution of the strain–time curves reflects different physical processes occurring during corrosion. During the initial stage of accelerated corrosion, the surface strain increased gradually as corrosion products accumulated at the steel–concrete interface and progressively filled the interfacial pores, generating compressive stresses that were subsequently transferred to the concrete surface. Once the corrosion-induced expansive stress exceeded the tensile strength of concrete, macrocracks initiated within the concrete cover. As these cracks propagated toward the concrete surface, the embedded optical fiber experienced significant tensile deformation, resulting in a sudden increase in the measured strain.

#### 3.1.2. Effect of Specimen Length on Concrete Surface Strain

**Figure 3:**
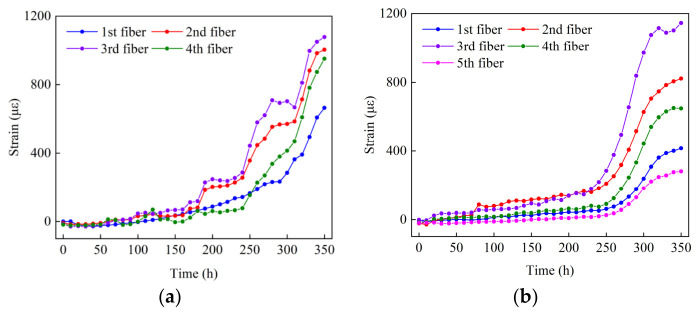

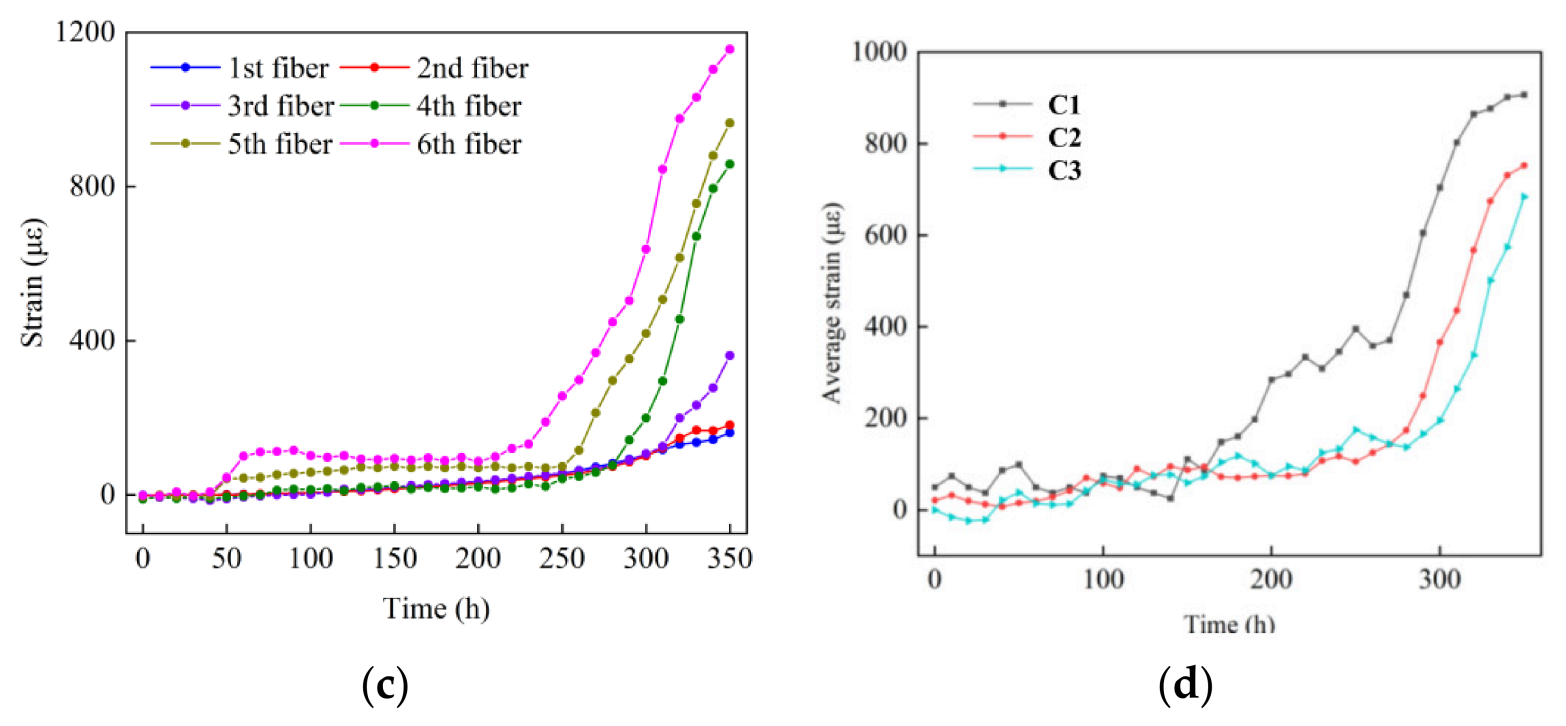
Optical fiber strain at the cracking location versus time for specimens (**a**) C1, (**b**) C2, and (**c**) C3; (**d**) average strain of each optical fiber embedded in concrete versus time for specimens with different lengths.

For specimens C1 (100 mm), C2 (300 mm), and C3 (500 mm), the ten sensing points located around the crack position on each optical fiber were selected, and their average strain values were calculated to obtain the corresponding strain–time curves, as shown in Figure 3a–c. Figure 3d compares the average strain evolution of all optical fibers for specimens with different lengths. Figure 3a–c clearly illustrate the differences in crack initiation time and the final strain level among the three specimen lengths. A common feature observed in all strain curves is that the strain increased gradually during the initial stage of accelerated corrosion and subsequently exhibited a sharp increase after reaching a critical point. This inflection point corresponds to the onset of concrete cracking, indicating the transition from the pore-filling and stress accumulation stage to the rapid propagation stage of macrocracks within the concrete cover.

Comparison of the strain–time histories demonstrates that the strain curves of the 100 mm specimens were relatively consistent among different optical fibers, with only minor differences in strain development. Crack initiation occurred within a relatively narrow time interval of approximately 200–250 h. In contrast, greater variability was observed in the 300 mm specimens. The third optical fiber entered the rapid strain-growth stage at approximately 250 h, whereas the remaining fibers did not exhibit a similar response until after approximately 270 h. The 500 mm specimens exhibited the most pronounced spatial heterogeneity. The third optical fiber showed a rapid increase in strain at approximately 220 h, considerably earlier than the first and sixth optical fibers, which entered the rapid strain-growth stage at approximately 280 h and 270 h, respectively. These observations indicate that increasing specimen length substantially amplified the differences in strain response among different locations. Figure 3d further shows that increasing specimen length resulted in a lower overall average strain. After approximately 160 h, the average strain of specimen C1 remained consistently higher than those of specimens C2 and C3, while the average crack initiation time was progressively delayed with increasing specimen length. These results demonstrate the characteristic behavior of high localized strain accompanied by a relatively low global average strain, indicating that corrosion-induced expansion in longer specimens became highly localized at a limited number of vulnerable regions, whereas the extensive inactive regions reduced the overall average strain through length averaging.

This phenomenon can be attributed primarily to the inherently random and spatially heterogeneous nature of reinforcement corrosion in concrete. As specimen length increased, the number of potential corrosion-active sites along the reinforcing steel also increased. Owing to local variations in concrete compactness, porosity, chloride concentration, and oxygen availability, these active sites exhibited different corrosion rates, resulting in multiple independently developing anodic regions. The interactions and competition among these anodic regions through the macro-cell corrosion effect produced a highly non-uniform distribution of corrosion products along the reinforcing steel surface [24]. Benefiting from its millimeter-scale spatial resolution, the distributed optical fiber sensing system effectively captured the localized strain concentrations and steep strain gradients induced by non-uniform corrosion. With increasing specimen length, the spatial variability of corrosion became progressively more pronounced, causing the mechanical response to evolve from synchronous deformation to asynchronous development. Consequently, significant differences in crack initiation time and peak strain were observed among different sensing locations. Furthermore, local defects in the concrete cover, entrapped air voids, or elevated chloride concentrations promoted earlier accumulation of corrosion products at vulnerable locations, enabling the corrosion-induced expansive stress to exceed the tensile strength of concrete and resulting in premature crack formation [25]. Therefore, specimen length not only influenced the magnitude and spatial variability of surface strain but also governed the temporal sequence of crack initiation at different locations, making it one of the key geometric parameters controlling corrosion-induced cracking.

#### 3.1.3. Effect of Biochar on Concrete Surface Strain

**Figure 4:**
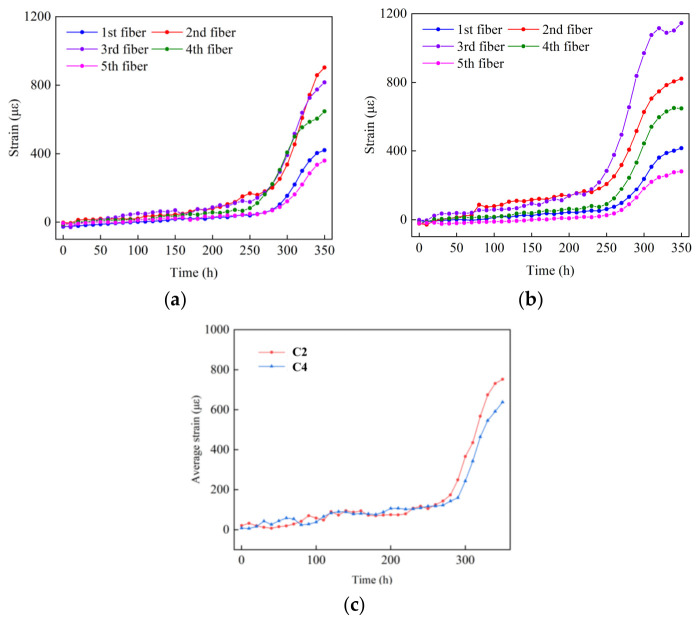
Optical fiber strain at the cracking location versus time for specimen (**a**) C4 and (**b**) C2; (**c**) average strain of each optical fiber embedded in concrete versus time for specimens C4 and C2.

Figure 4a,b present the strain–time histories of all optical fibers located near the crack positions in specimen C4 (0.5 wt.% biochar) and the reference specimen C2, respectively, whereas Figure 4c compares the average strain evolution of all optical fibers in the two specimens. As shown in Figure 4a,b, the strain curves of specimen C2 were relatively scattered. Some optical fibers, such as the 3rd fiber, exhibited a rapid increase in strain at approximately 250 h, whereas others, such as the 5th fiber, responded noticeably later, indicating pronounced spatial heterogeneity in crack initiation. In contrast, the strain curves of specimen C4 were more closely grouped, exhibiting a lower strain growth rate and improved synchronization among different optical fibers. As shown in Figure 4c, the average strain of specimen C4 remained consistently lower than that of specimen C2 throughout the monitoring period. The rapid strain-growth stage occurred considerably later than that of the reference specimen. At 350 h, the average strain of specimen C4 was approximately 645 με, representing a reduction of approximately 17% compared with specimen C2 (776 με). These results indicate that biochar incorporation not only delayed the rapid development of surface strain and reduced the final strain level but also effectively mitigated the spatial heterogeneity of corrosion-induced strain.

The beneficial effect of biochar on suppressing surface strain can be attributed primarily to its unique porous structure and physicochemical properties. Owing to its high specific surface area (900–1300 m^2^/g) and abundant microporous structure, biochar effectively adsorbed chloride ions, thereby reducing the local chloride concentration around the reinforcing steel and delaying passive film breakdown as well as corrosion initiation [26,27]. Furthermore, the porous structure of biochar served as a temporary storage reservoir for corrosion products during the early corrosion stage, delaying the completion of pore filling at the steel–concrete interface and consequently postponing the rapid accumulation of corrosion-induced expansive stress. In addition, biochar incorporation improved the pore structure of concrete, reduced capillary water absorption, and enhanced matrix compactness, thereby restricting the transport of chloride ions and oxygen. These two effects occur at different structural scales. The internal micropores of biochar particles provide localized storage space for corrosion products, whereas the overall concrete matrix becomes denser because biochar particles fill voids and improve the interfacial transition zone. Therefore, biochar simultaneously provides internal adsorption/storage sites while reducing continuous transport pathways for chloride ions and oxygen.

Beyond suppressing the average strain, the mitigation of strain heterogeneity deserves further explanation. Biochar does not create a perfectly uniform strain field—which is physically unattainable—but rather makes the distribution relatively more homogeneous. This is achieved through three interrelated effects. First, biochar adsorbs free chlorides and modulates local concentration gradients, reducing spatial fluctuations of the chloride front and dispersing corrosion hot spots. Second, unlike biochar-free systems where chlorides cause highly localized pitting along defects, biochar promotes wider dispersion of initiation sites, so that rust expansion is spread over a larger area, lowering peak local strains. Third, this is quantitatively evidenced in Figure 4a,b: the standard deviation and range of strain values among different fibers are markedly reduced in specimen C4, indicating that the dispersion of strain distribution—not just the mean—is significantly decreased.

Through the combined action of these mechanisms, biochar reduced the corrosion activity and chloride penetration rate at different locations, leading to more uniform strain evolution among different optical fibers and a more synchronized cracking process [26].

#### 3.1.4. Effect of Concrete Cover Thickness on Surface Strain

**Figure 5:**
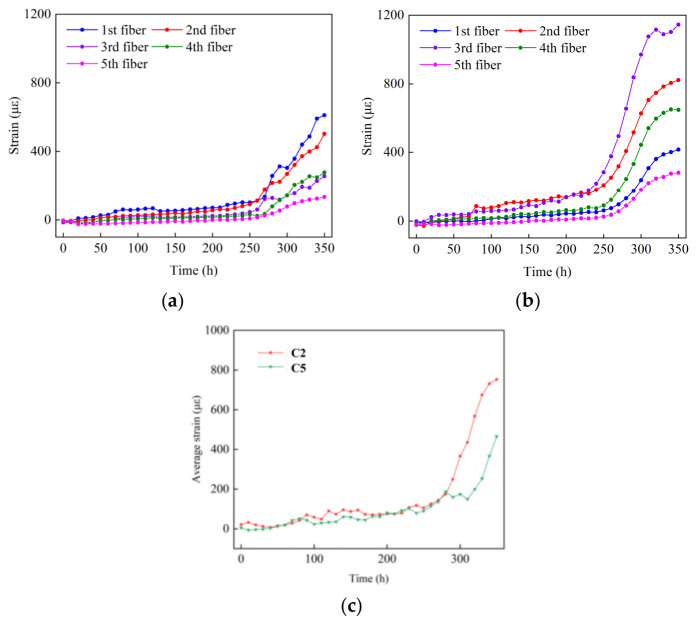
Optical fiber strain at the cracking location versus time for specimen (**a**) C5 and (**b**) C2; (**c**) average strain of each optical fiber embedded in concrete versus time for specimens C5 and C2.

Figure 5a,b show the strain–time histories of all optical fibers located near the crack positions in specimens C5 (40 mm concrete cover) and C2 (25 mm concrete cover), respectively, whereas Figure 5c compares the average strain evolution of all optical fibers for the two cover thicknesses. As illustrated in Figure 5a,b, the strain curves of specimen C2 were relatively scattered, with some optical fibers exhibiting a rapid strain increase at approximately 250 h, whereas others responded later, indicating pronounced spatial heterogeneity in crack initiation. In contrast, the strain curves of specimen C5 were smoother and exhibited more consistent strain growth rates, suggesting a more synchronized damage evolution among different optical fibers. Figure 5c further shows that the average strain of the 40 mm cover specimen remained consistently lower than that of the 25 mm cover specimen throughout the monitoring period. The onset of rapid strain development was delayed from approximately 250 h to more than 300 h. At 350 h, the average strain of specimen C5 was approximately 471 με, representing a reduction of approximately 39% compared with specimen C2 (776 με). Among all investigated parameters, increasing the concrete cover thickness exhibited the most significant suppression of strain development. These results indicate that increasing the concrete cover thickness not only delayed crack initiation but also effectively reduced the spatial heterogeneity of corrosion-induced strain.

The beneficial effect of increasing the concrete cover thickness can be explained in two aspects. Under the electrically accelerated corrosion conditions adopted in this study, chloride transport was primarily governed by the applied electric field. Increasing the concrete cover thickness substantially extended the transport path of chloride ions, thereby delaying their arrival at the reinforcing steel surface and postponing passive film breakdown as well as corrosion initiation [28]. Meanwhile, a thicker concrete cover provided greater circumferential confinement against corrosion-induced expansion, resulting in lower circumferential tensile stress within the concrete cover under the same radial expansion displacement. Furthermore, the longer crack propagation path promoted greater energy dissipation through crack surface friction and concrete plastic deformation, thereby reducing the net strain transmitted to the concrete surface [29]. In addition, the thicker concrete cover provided stronger residual confinement after local cracking, delaying the rapid ingress of aggressive agents through cracks and consequently suppressing the positive feedback effect associated with corrosion propagation [30].

#### 3.1.5. Effect of Reinforcing Bar Diameter on Concrete Surface Strain

**Figure 6:**
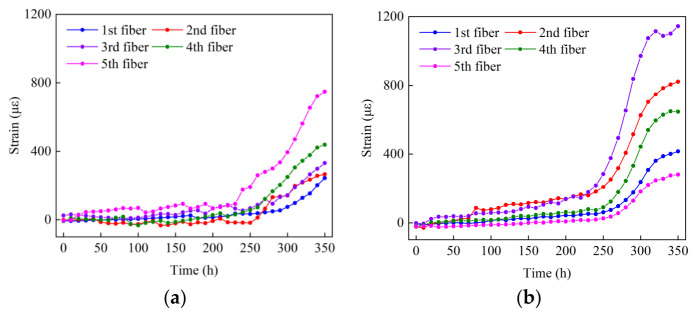

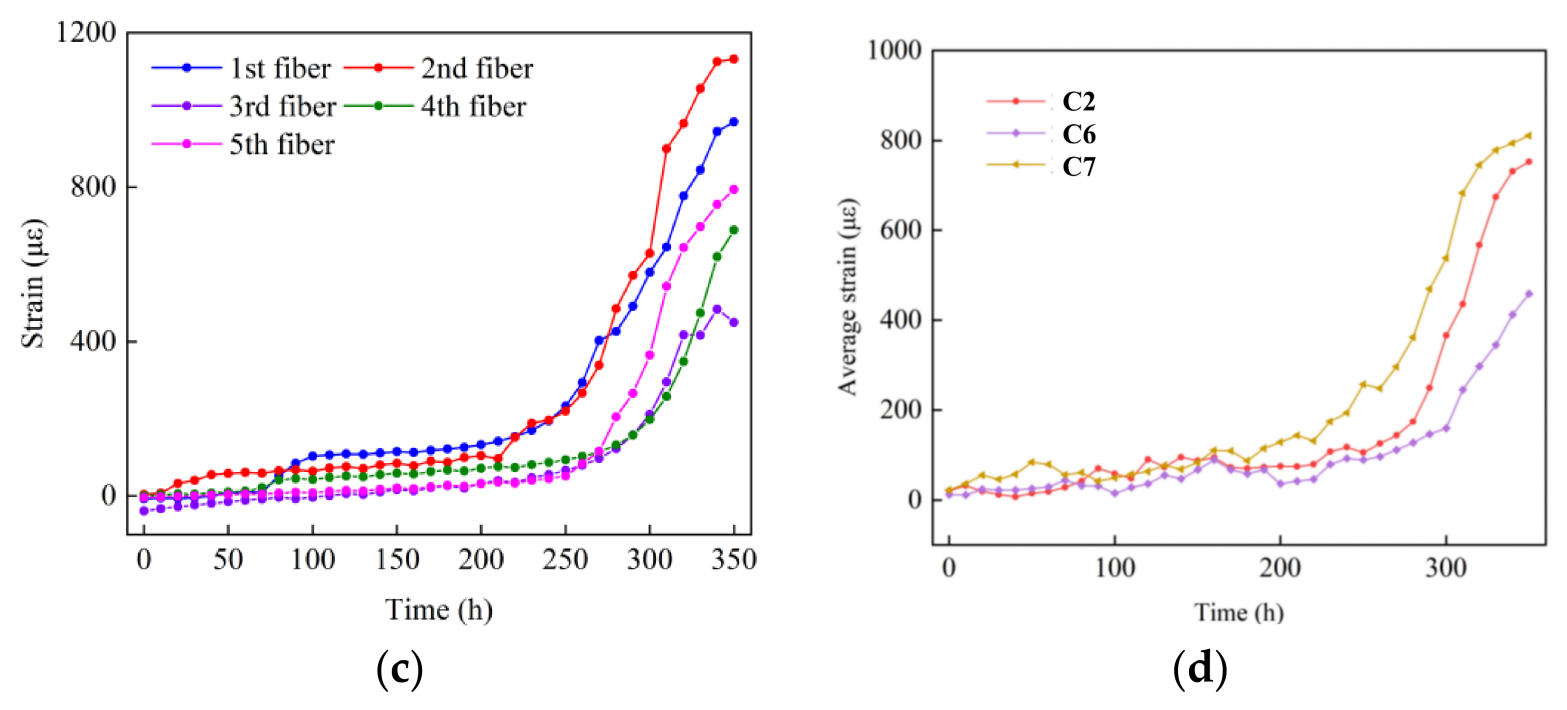
Optical fiber strain at the cracking location versus time for specimen (**a**) C6, (**b**) C2 and (**c**) C7; (**d**) average strain of each optical fiber embedded in concrete versus time for specimens C6, C2, and C7.

A comparison of the strain–time histories presented in Figure 6a–c shows that the strain curves of all optical fibers in the R = 12 mm specimens almost completely overlapped, exhibiting minimal variation among different sensing locations. All sections entered the rapid strain-growth stage nearly simultaneously between 200 h and 250 h. For the R = 20 mm specimen, the strain curves were generally consistent during the early stage of corrosion but gradually diverged after entering the rapid strain-growth stage, with some optical fibers exhibiting strain surges at approximately 200 h, whereas others responded noticeably later. In contrast, the R = 25 mm specimens exhibited two distinct groups of strain curves. Two optical fibers entered the rapid strain-growth stage at approximately 200 h, whereas the remaining three optical fibers exhibited significantly delayed crack initiation. These observations indicate that increasing the reinforcing bar diameter intensified the spatial heterogeneity of corrosion-induced expansion, making certain locations more susceptible to premature cracking. As shown in Figure 6d, increasing the reinforcing bar diameter accelerated the development of average strain and resulted in higher final strain values. The R = 12 mm specimens exhibited the slowest strain growth, with rapid strain development beginning at approximately 250 h and reaching an average strain of approximately 431 με at 350 h. The corresponding values for the R = 20 mm specimens were approximately 250 h and 776 με, whereas the R = 25 mm specimens entered the rapid strain-growth stage at approximately 200 h and exhibited the highest final average strain of approximately 817 με.

The promoting effect of reinforcing bar diameter on surface strain development can be explained from two aspects. Under identical electrically accelerated corrosion conditions, the total mass of corrosion products generated per unit area of reinforcing steel was approximately equivalent. However, when the same amount of corrosion products accumulated on reinforcing bars with different diameters, larger reinforcing bars produced greater radial expansion displacement owing to their larger radius, thereby generating a stronger corrosion-induced expansive force [31]. Moreover, because the concrete cover thickness remained constant at 25 mm, increasing the reinforcing bar diameter reduced the relative concrete cover thickness (c/d), decreasing from approximately 2.08 for R = 12 mm to 1.25 for R = 20 mm and 1.00 for R = 25 mm. A lower c/d ratio reduced the circumferential confinement provided by the concrete cover, thereby facilitating crack initiation and propagation [32].

In addition, corrosion of steel in concrete is inherently non-uniform; chlorides preferentially attack local anodic sites, leading to pitting rather than uniform dissolution. For larger-diameter reinforcement, although the increased surface area may provide a somewhat greater number of potential initiation sites, the key point is that the significantly larger total amount of rust does not spread evenly over the extra area. Instead, the additional corrosion products are still predominantly concentrated at these localized regions. As a result, the local volumetric expansion at each active site is considerably greater than that in smaller-diameter bars. This intensified local accumulation generates higher radial expansive pressure against the surrounding concrete, which in turn induces larger circumferential tensile stresses in the cover zone, ultimately manifesting as higher surface strain and more pronounced cracking.

These two mechanisms jointly explain why larger reinforcing bar diameters resulted in earlier strain development, higher strain levels, and more pronounced spatial heterogeneity, reflecting the combined effects of increased corrosion-induced expansive force and reduced confinement efficiency.

### 3.2. Effect of the Spatial Heterogeneity of Chloride Concentration Distribution on Concrete Cracking

To quantitatively elucidate the relationship between the spatial distribution of chloride ions and corrosion-induced cracking of concrete, concrete powder samples were collected from the upper surface directly above the reinforcing steel after completion of the accelerated corrosion test. Along the longitudinal direction of each specimen, the concrete was divided into 10 equal segments, and one powder sample was drilled from the center of each segment. The segment lengths were 1 cm, 3 cm, and 5 cm for the 100 mm, 300 mm, and 500 mm specimens, respectively. The free chloride concentration at each sampling location was subsequently determined using the water-soluble extraction method. The chloride concentration distributions were then compared with the strain measured by the distributed optical fibers at the corresponding crack locations on the concrete surface.

**Figure 7:**
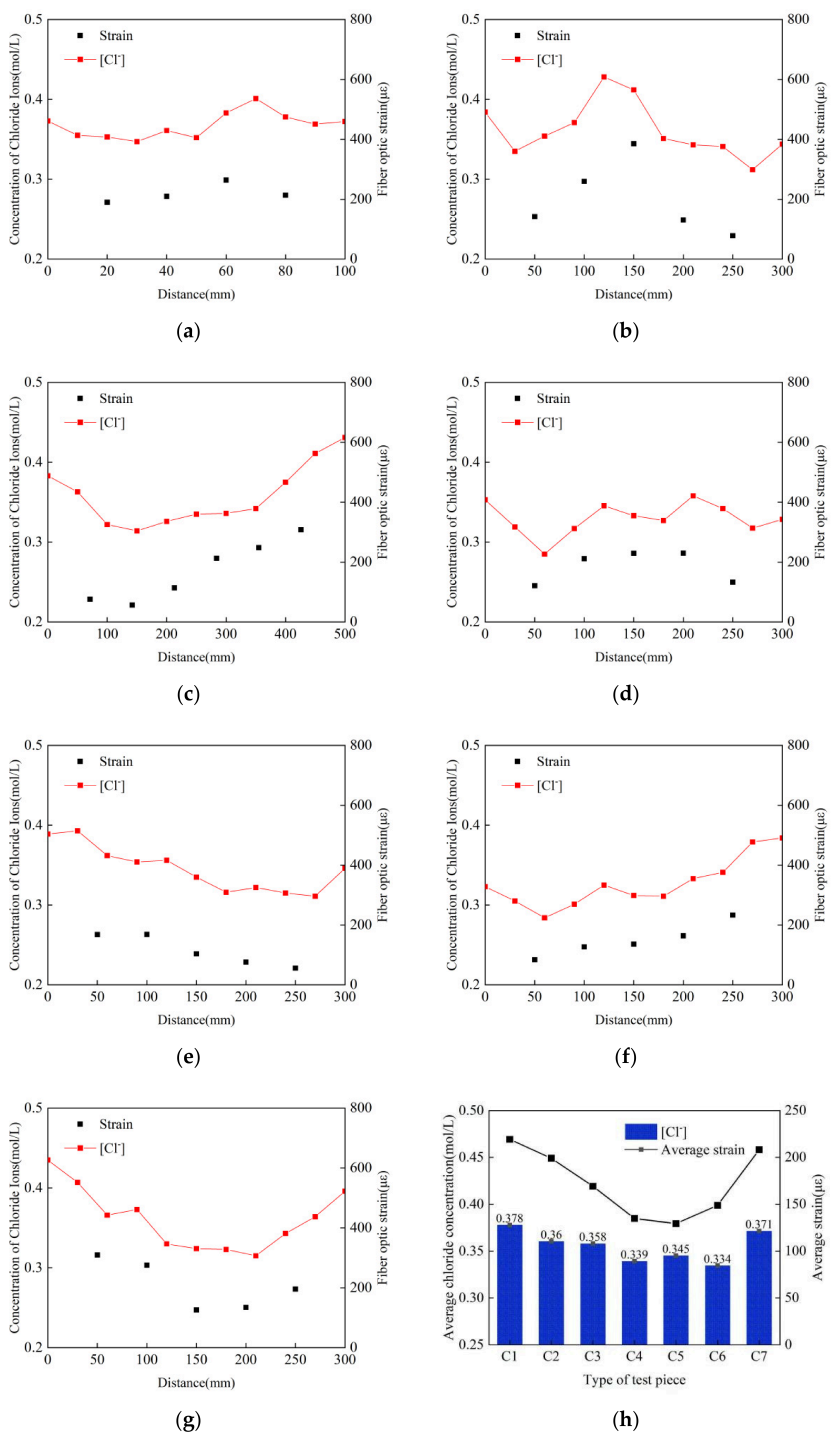
(**a**–**g**) Comparison of the spatial distributions of chloride concentration on the reinforcing bar surface and the corresponding cracking strain for specimens C1–C7, respectively; (**h**) comparison of the average strain and the average chloride concentration during the cracking stage of the specimens.

Figure 7a–g present the longitudinal distributions of free chloride concentration together with the corresponding crack strain for specimens C1–C7, respectively, whereas Figure 7h summarizes the average free chloride concentration and the corresponding average surface strain during the cracking stage for all specimens. As shown in Figure 7h, the average chloride concentration exhibited an overall positive correlation with the average surface strain during the cracking stage, indicating that chloride concentration is one of the primary factors governing reinforcement corrosion and the subsequent cracking of concrete. Higher chloride concentrations accelerate the breakdown of the passive film on the reinforcing steel, thereby promoting the formation of corrosion products and increasing the corrosion-induced expansive stress. However, chloride concentration was not the sole factor controlling the magnitude of cracking. A comparison between the reference specimen C2 and specimen C5 with a 40 mm concrete cover shows that the average free chloride concentration of C5 was only approximately 3.7% lower than that of C2, whereas the corresponding average strain decreased by approximately 30%. This pronounced discrepancy indicates that, even under similar levels of chloride ingress, the mechanical restraint provided by the concrete plays a critical role in governing the surface strain response. The concrete cover thickness and reinforcing bar diameter jointly determine the circumferential confinement efficiency of the concrete cover. A higher confinement efficiency results in lower circumferential tensile stress within the concrete cover under the same corrosion-induced expansion displacement [29,30]. Therefore, the chloride concentration primarily determines the driving force for corrosion-induced expansion, whereas the confinement capacity of the concrete governs the structural response to this expansive force. The combined effects of these two factors ultimately control the initiation and development of corrosion-induced cracking.

### 3.3. Estimation of Crack Width at the Optical Fiber Location

**Figure 8:**
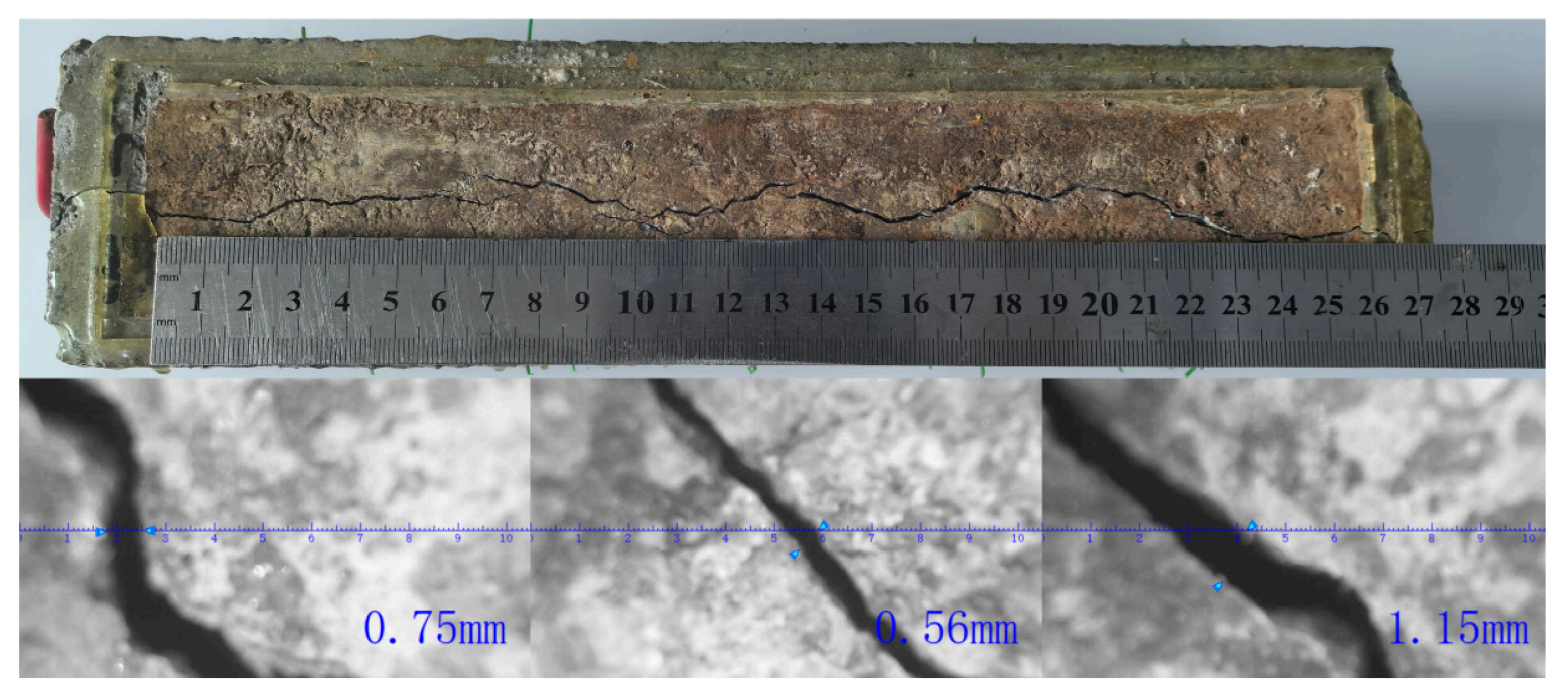
Measurement and location of concrete cracks. (The distance between two blue arrows were measured as the crack width in the figure).

As the constant-current accelerated corrosion test progressed, longitudinal cracks parallel to the reinforcing steel gradually appeared on the upper surface of the concrete specimens after approximately 250 h. Subsequently, the crack width continuously increased with corrosion duration, while the number of cracks progressively increased and the cracks propagated laterally. At 350 h, multiple surface cracks interconnected to form a dominant through-crack with a maximum width of approximately 0.8 mm. For each specimen, the widest crack located at the optical fiber installation position was selected for subsequent analysis. The crack measurement procedure is illustrated in Figure 8.

#### 3.3.1. Analysis of Crack Width

The measured crack width was obtained by directly measuring the maximum crack opening at each crack location on the upper concrete surface. Each measurement point was measured three times, and the arithmetic mean was adopted as the final measured crack width.

The crack-width estimation based on the optical fiber strain integration method is founded on the principle that the total elongation of the optical fiber can be obtained by spatially integrating the measured strain along the sensing path [33,34]. For an individual crack, the crack width can be calculated using Equation (1)

(2) $$\omega_{i}=\int_{x_{i,1}}^{x_{i,2}}\varepsilon \left(x\right)dx$$

where *w_i_* denotes the width of the i-th crack; *x*_*i*,1_ and *x*_*i*,2_ represent the start and end positions of the influence zone of the i-th crack, respectively; and *ε*(x) is the strain distribution measured along the optical fiber in the x-direction.

**Figure 9:**
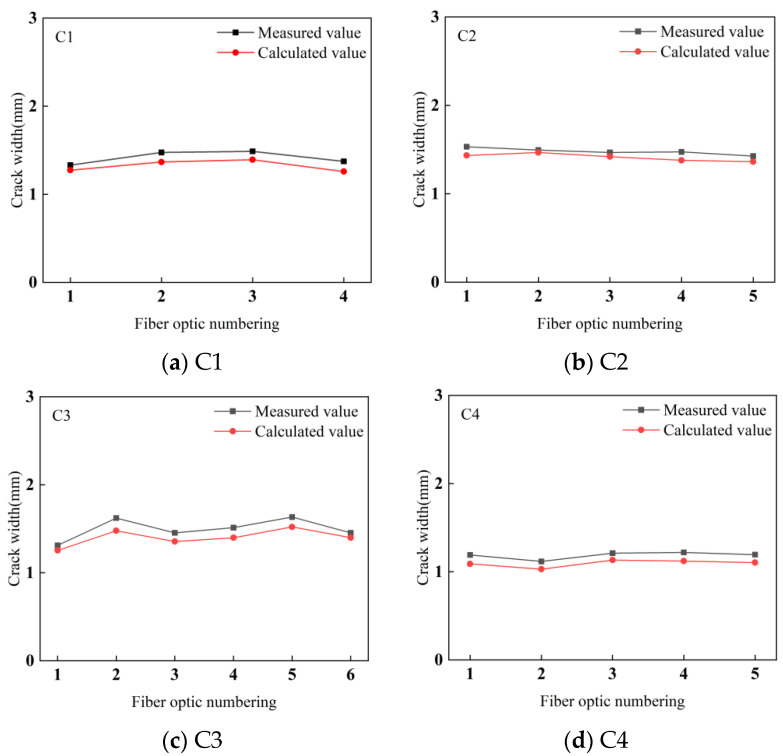

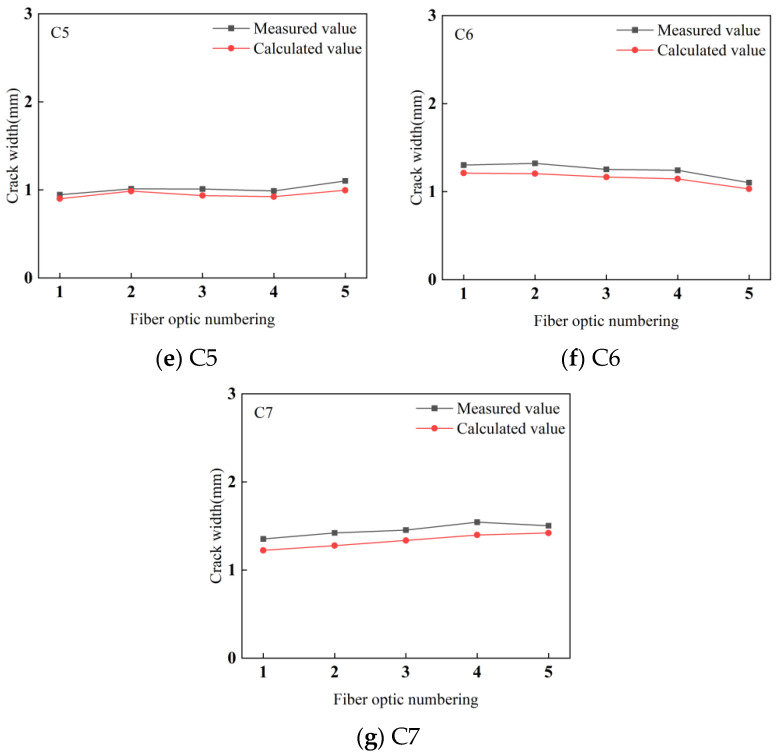
Comparison of calculated and measured values of crack width for specimens (**a**–**g**): C1–C7.

Within the crack influence zone, the optical fiber underwent tensile deformation synchronously with the opening of the concrete surface crack, and the total elongation of the optical fiber corresponded to the crack opening width at that location. In this study, the strain data in the vicinity of each crack were extracted, and the theoretical crack width at each crack location was obtained by applying the trapezoidal numerical integration method based on Equation (1). The measured crack widths at all monitoring locations were then compared with the corresponding calculated values obtained from strain integration, as presented in Figure 9. The measured and theoretical crack widths exhibited generally consistent evolution trends for all specimens, indicating that the optical fiber strain integration method demonstrates good agreement in characterizing the relative spatial distribution of crack widths. Considering all measurement locations, the average measured crack width was 1.21 mm, whereas the average calculated crack width obtained from optical fiber strain integration was 1.15 mm, corresponding to a difference of 0.06 mm and a relative deviation of approximately 5%. Nevertheless, the measured crack widths were consistently greater than the corresponding theoretical values at almost all measurement locations, indicating the existence of a systematic positive deviation between the two methods.

The systematic deviation can be primarily attributed to the following two factors. First, uncertainty exists in determining the integration interval. In practice, the boundaries of the crack influence zone cannot be identified precisely, and microcracked regions with gradually decreasing strain are generally present on both sides of the strain peak. Consequently, the selection of the integration start and end points inevitably involves a certain degree of subjectivity, which may lead to an integration interval that is shorter than the actual crack influence zone and neglect part of the strain contribution from the boundary regions, thereby resulting in an underestimation of the calculated crack width. Second, Discrepancies exist between the actual crack geometry and the optical fiber path. In reality, cracks propagate in a tortuous or irregular three-dimensional manner and are not necessarily perpendicular to the optical fiber. In contrast, the strain integration is performed along a straight optical fiber path, and the accumulated elongation represents only the projection of the crack opening displacement onto the optical fiber axis. Therefore, when the crack surface is inclined or exhibits a tortuous propagation path, the projected displacement measured by the optical fiber may be smaller than the actual crack opening width [35,36]. The combined effects of these two factors result in theoretical crack widths obtained from strain integration being systematically lower than the corresponding experimentally measured values.

#### 3.3.2. Evolution of Crack Width with Time

**Figure 10:**
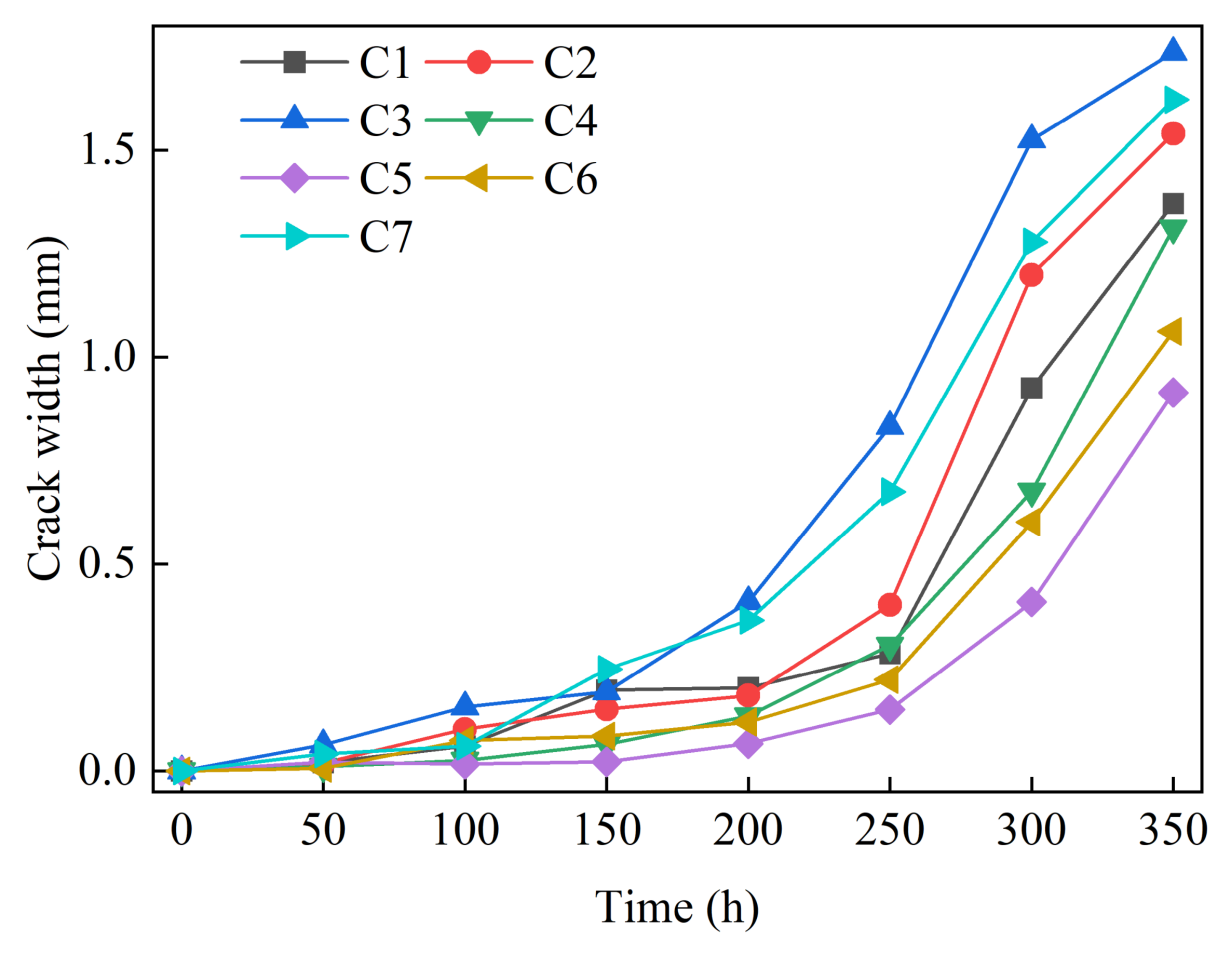
Calculated crack width versus time.

The strain data obtained from each optical fiber were integrated to calculate the temporal evolution of the theoretical crack width for each specimen, as shown in Figure 10. The crack width of all specimens exhibited an overall increasing trend with increasing corrosion duration. However, the crack growth rates varied significantly among the different specimens. The reference specimen (C2) exhibited the highest crack growth rate, reaching a theoretical crack width of approximately 1.15 mm after 350 h of accelerated corrosion. In contrast, specimens C4 (biochar), C5 (40 mm concrete cover), and C6 (R = 12 mm) exhibited significantly lower crack growth rates than C2, indicating that biochar incorporation, increasing the concrete cover thickness, and reducing the reinforcing bar diameter were all effective in delaying crack propagation. By comparison, specimens C3 (500 mm in length) and C7 (R = 25 mm) exhibited higher crack growth rates than C2, which is consistent with the strain evolution characteristics presented in Section 3.1.

## 4. Conclusions

In this study, a distributed optical fiber sensing (DOFS) system was employed to perform continuous, high-resolution in situ monitoring of the surface strain evolution and crack development of reinforced concrete specimens subjected to electrically accelerated corrosion. By comparatively investigating the effects of specimen length, biochar incorporation, concrete cover thickness, and reinforcing bar diameter, the spatiotemporal evolution characteristics of corrosion-induced surface strain and the non-uniform cracking behavior of concrete were systematically revealed. The main conclusions are summarized as follows:

(1) The distributed optical fiber sensing technique successfully captured the continuous evolution of concrete surface strain under non-uniform corrosion-induced expansion with high spatial resolution. The strain–time curves exhibited a characteristic two-stage evolution consisting of an initial slow-growth stage followed by a rapid-growth stage, corresponding to the pore-filling and stress accumulation process during the early corrosion stage and the subsequent initiation and penetration of macrocracks, respectively. This evolution pattern provides a clear stage-based criterion for the early warning of corrosion-induced cracking.

(2) Increasing the specimen length significantly amplified the spatiotemporal heterogeneity of corrosion-induced strain, resulting in the characteristic distribution of high localized strain peaks accompanied by relatively low average surface strain. A longer specimen provided more potential corrosion-active sites along the reinforcing steel, causing the initiation time of the rapid cracking stage at different optical fiber locations to differ by more than 50 h. Consequently, the strain response evolved from synchronous development to asynchronous evolution.

(3) Incorporation of 0.5 wt.% biochar reduced the average surface strain by approximately 17% and delayed crack initiation to approximately 260 h. This improvement can be attributed to the physical adsorption and chemical immobilization of chloride ions by the high specific surface area of biochar, together with its ability to accommodate corrosion products within its porous structure during the early corrosion stage. These mechanisms effectively reduced the driving force for corrosion-induced expansion and suppressed the spatial heterogeneity of strain development.

(4) Increasing the concrete cover thickness exhibited the most pronounced inhibitory effect on corrosion-induced cracking, reducing the average surface strain by approximately 39% and delaying crack initiation to approximately 320 h, whereas reducing the reinforcing bar diameter decreased the peak strain to approximately 79% of that of the reference specimen. Although these two parameters differ in structural configuration, they share the same fundamental mechanical mechanism, namely enhancing the circumferential confinement efficiency of concrete by increasing the relative concrete cover thickness (c/d). In addition, increasing the concrete cover thickness provided an extra chemical retardation effect by extending the chloride transport path.

(5) The crack widths estimated from optical fiber strain integration exhibited good agreement with the experimentally measured crack widths in terms of their overall evolution trend, demonstrating the validity of the proposed method. The theoretical crack widths obtained from strain integration were slightly lower than the measured values, with a relative deviation of approximately 5%. This discrepancy was mainly attributed to uncertainties in determining the integration interval and to the geometric mismatch between the three-dimensional crack morphology and the one-dimensional optical fiber sensing path.

(6) Chloride concentration is an important factor driving reinforcement corrosion and the subsequent cracking of concrete; however, it is not the sole factor governing crack development. The crack initiation locations were strongly influenced by the macro-cell corrosion effect induced by abrupt chloride concentration gradients, where regions with severe chloride concentration gradients preferentially developed localized anodic zones and experienced accelerated corrosion. Meanwhile, the circumferential confinement efficiency of concrete played a critical role in regulating the strain response. Therefore, chloride concentration and confinement efficiency jointly constitute the primary controlling factors governing corrosion-induced cracking.

## Figures and Tables

**Table 1:** Chemical compositions (%) of Portland cement.

CaO	Al_2_O_3_	SiO_2_	Fe_2_O_3_	MgO	SO_3_	TiO_2_	Loss
57.34	6.73	27.82	4.36	3.32	3.7	0.56	4.77

**Table 2:** Chemical compositions of artificial seawater.

NaCl	MgCl_2_	Na_2_SO_4_	CaCl_2_	KCl	NaHCO_3_	KBr
24.53 g/L	5.20 g/L	4.09 g/L	1.16 g/L	0.695 g/L	0.201 g/L	0.101 g/L

**Table 3:** Biochar parameters.

Material	Model	Granularity	Bulk Density (g/L)	Specific Surface Area (m^2^/g)	pH	Carbon Content (%)
Sawdust biochar	SC-102	200	400–450	900–1300	4–11	≥95%

**Table 4:** Dimensions of test specimens and concrete mix proportion.

Test-Piece No.	Specimen Size (mm)	Cementitious Material	Rebar Diameter (mm)	Mix Proportion (kg/m^3^)	Mix Proportion
C1	70 × 70 × 100	Cement	R = 20	Cement:Sand:Water:Superplasticizer = 500:1250:205:5	0.41
C2	70 × 70 × 300	Cement	R = 20	Cement:Sand:Water:Superplasticizer = 500:1250:205:5	0.41
C3	70 × 70 × 500	Cement	R = 20	Cement:Sand:Water:Superplasticizer = 500:1250:205:5	0.41
C4	70 × 70 × 300	99.5%Cement + 0.5%Sawdust	R = 20	Cement:Biochar:Sand:Water:Superplasticizer = 497.5:2.5:1250:205:5	0.41
C5	100 × 100 × 300	Cement	R = 20	Cement:Sand:Water:Superplasticizer = 500:1250:205:5	0.41
C6	62 × 62 × 300	Cement	R = 12	Cement:Sand:Water:Superplasticizer = 500:1250:205:5	0.41
C7	75 × 75 × 300	Cement	R = 25	Cement:Sand:Water:Superplasticizer = 500:1250:205:5	0.41

## Data Availability

Data will be made available upon request.
